# Comparison of clinical outcomes between cone beam CT-guided thermal ablation and helical tomotherapy in pulmonary metastases from hepatocellular carcinoma

**DOI:** 10.3389/fonc.2022.947284

**Published:** 2022-08-17

**Authors:** Feihang Wang, Shaonan Fan, Qin Shi, Danyang Zhao, Huiyi Sun, Yav Sothea, Mengfei Wu, Huadan Song, Yi Chen, Jiemin Cheng, Zhaochong Zeng, Zhiping Yan, Jian He, Lingxiao Liu

**Affiliations:** ^1^ Department of Interventional Radiology, Zhongshan Hospital, Fudan University, Shanghai, China; ^2^ Shanghai Institute of Medical Imaging, Fudan University, Shanghai, China; ^3^ National Clinical Research Center for Interventional Medicine, Zhongshan Hospital, Fudan University, Shanghai, China; ^4^ Department of Radiation Oncology, Zhongshan Hospital, Fudan University, Shanghai, China; ^5^ Shanghai Medical College, Fudan University, Shanghai, China; ^6^ Department of Computed Tomography (CT) and Magnetic Resonance Imaging (MRI), Third Hospital of Hebei Medical University, Shijiazhuang, China; ^7^ Department of Radiology, Xinhua Hospital, Shanghai Jiao Tong University School of Medicine, Shanghai, China

**Keywords:** pulmonary metastases, hepatocellular carcinoma, thermal ablation, helical tomotherapy, comparison

## Abstract

**Objective:**

This retrospective study compares the clinical results of cone beam CT (CBCT)-guided thermal ablation with those of helical tomotherapy in hepatocellular carcinoma (HCC) patients with pulmonary metastases.

**Methods:**

A total of 110 patients undergoing thermal ablation or helical tomotherapy for pulmonary metastases from April 2014 to December 2020 were included in the study. The endpoints were local tumor progression-free survival (LTPFS), overall survival (OS), and complications. Univariate and multivariate analyses using the Cox proportional hazard model were conducted to identify independent factors (univariate: *P* < 0.1; multivariate: *P* < 0.05). The Kaplan–Meier method was used to calculate the LTPFS and OS rates.

**Results:**

The results of 106 patients were taken into the final analysis. The 1- and 3-year LTPFS rates were 50 and 19% for the thermal ablation group and 65 and 25% for the helical tomotherapy group. The median LTPFS in the thermal ablation group was 12.1 months, while it was 18.8 months in the helical tomotherapy group (*P* = 0.25). The 1- and 3-year OS rates were 75 and 26% for the thermal ablation group and 77 and 37% for the helical tomotherapy group. The median OS was 18.0 months in the thermal ablation group and 23.4 months in the helical tomotherapy group (*P* = 0.38). The multivariate analyses found that α-fetoprotein (AFP) at <400 ng/ml (*P* = 0.003) was significantly associated with better LTPFS. Tumor number <3 and AFP <400 ng/ml were favorable prognostic factors for OS. There were no grades 3–5 adverse events in both groups. Grade 2 was recorded in three patients (4.8%) in the thermal ablation group and two patients (4.7%) in the helical tomotherapy group.

**Conclusions:**

For pulmonary metastases from HCC, CBCT-guided thermal ablation and helical tomotherapy provided comparable clinical effects and safety.

## Introduction

The lung is the most common site of extrahepatic metastases in patients with hepatocellular carcinoma (HCC) (1). Systemic therapy is the mainstream treatment for HCC patients with extrahepatic metastases (2). Sorafenib is recommended as the first-line therapy, and its median overall survival (OS) was 7.13–9.6 months for HCC patients with extrahepatic metastases (3, 4). However, a phase 2 trial revealed that pulmonary metastases had a poor response to sorafenib in advanced HCC patients (5). Surgery is the mainstream of locoregional treatment for pulmonary metastases (6). In 2018, a meta-analysis reported that the median OS of patients with pulmonary metastases from colorectal carcinoma undergoing pulmonary metastasectomy was 43 months (7). The 5-year survival rate for pulmonary metastasectomy of HCC was 66.9 ± 10% (8). However, most patients are not suitable for surgery due to poor liver function that is unable to tolerate surgery, surgical trauma, and multiple or bilateral metastases of pulmonary metastases (9, 10).

Noninvasive or minimally invasive locoregional treatments, such as thermal ablation and radiotherapy, have gained increasing acceptance (11). In China, guidelines and expert consensus demonstrated that thermal ablation and radiotherapy could be used as curative or palliative therapy for treating pulmonary metastases from hepatocellular carcinoma (12–14). A previous study compared the effectiveness of thermal ablation and stereotactic radiotherapy in lung cancer and reported no significant overall survival difference between the two therapies (*P* = 0.13) (15). In HCC patients with pulmonary metastases, thermal ablation could acquire 79.8% 1-year OS and 58% 3-year OS (16), and radiotherapy could achieve 65.5% 1-year OS (17). Nevertheless, there is a lack of direct comparisons of clinical outcomes between thermal ablation and radiotherapy. This retrospective study directly compares the clinical outcomes between thermal ablation and helical tomotherapy in HCC patients with pulmonary metastases.

## Materials and methods

### Patients

This retrospective study was approved by the institutional review boards of Zhongshan Hospital, Fudan University. Informed consent was waived. From April 2014 to December 2020, 110 patients with lung metastasis from liver cancer, who underwent thermal ablation or helical tomotherapy for lung metastasis, were selected for this study. The inclusion criteria were as follows: (a) age between 18 and 80 years, (b) clinical or histological diagnosis of lung metastasis from liver cancer, (c) controlled intrahepatic tumors, and (d) patients refused or were not suitable for lung metastasectomy. The exclusion criteria were as follows: (a) liver cancer was diagnosed as intrahepatic cholangiocarcinoma (ICC), (b) uncorrectable coagulopathy, and (c) loss to follow-up after treatment or incomplete medical records.

Chest computed tomography (CT) or ^18^F-fluorodeoxyglucose positron emission tomography-CT was used for pretreatment evaluation. Laboratory tests including blood routine, coagulation function, liver function, and alpha-fetoprotein were also performed.

### Cone beam CT-guided thermal ablation

**Figure 1:**
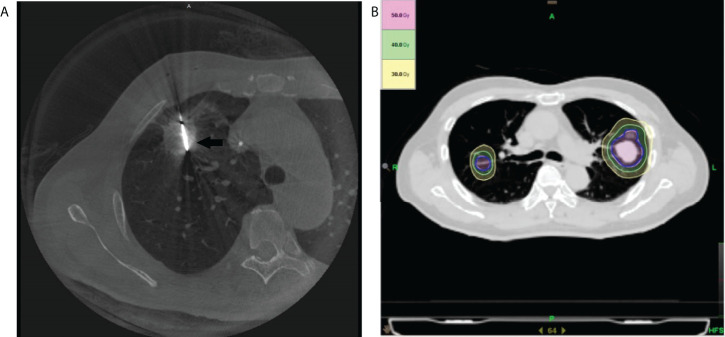
Illustration of the treatment plan. **(A)** The axial cone beam CT image obtained during microwave ablation shows a microwave antenna positioned in the tumor (arrow). **(B)** An SBRT treatment plan with isodose curve distribution for a patient with bilateral pulmonary metastasis of hepatocellular carcinoma is displayed.

The patients were scanned by cone beam CT (CBCT) to determine the puncture angles, depths, and appropriate positions. After the scan, local anesthesia was administrated with 1% lidocaine at the selected puncture point. Then, a 17-gauge trocar was inserted into the target tumor to guide the core-needle biopsy, antenna, or electrode to the target. For microwave ablation (MWA), the antenna was advanced into the target tumor, and the power was set at 40-60W (**Figure 1A**). For radiofrequency ablation (RFA), the electrode was inserted into the target lesion, and radiofrequency energy was applied with an impedance control algorithm for 8-14 minutes. At the end of the MWA or RFA procedure, CBCT scans were performed to confirm that the ablation margin around the tumor was more than 5 mm and to evaluate complications. The needle track was also ablated to avoid bleeding and tumor seeding along the electrode route.

### Helical tomotherapy

All treatments were administered using Helical Tomotherapy (HT) Hi-Art Treatment System (Accuray, Madison, WI, USA). Radiation therapy (RT) was delivered using either intensity-modulated radiation therapy (IMRT) or SBRT based on tumor location, tumor size, and physician/patient preference. Radiotherapy simulation was done in the supine position with both arms overhead using a vacuum bag. All patients were simulated with the application of respiration-correlated helical four-dimensional CT (4D-CT) (Siemens Somatom Sensation, Siemens Healthineers Corporation, Germany) scans with a slice thickness of 3 mm (**Figure 1B**).

The gross tumor volume (GTV) was delineated as a lesion observed at the lung window level on the enhanced CT and/or PET/CT. The clinical target volume was equal to gross tumor volume. The internal target volume (ITV) was contoured based on the extension of GTVs at all phases (five inspiratory, five expiratory, and one resting) of the respiratory cycle on the 4D-CT scanning to include the full movement of the tumor. To compensate for uncertainty of the tumor position and changes of the tumor motion caused by breathing, the planning target volume extended a margin of 0.5 cm from the ITV. Cone beam CT was implemented before each treatment to confirm that the position of the target was achieved.

The HT-SBRT technique and treatment planning were performed as previously described according to our institutional protocol (1): In general, the fractionation regimes primarily depended on the treating physicians’ preference, based on tumor location, tumor size, and lung function parameters. Typically, 5.0 to 10.0 Gy per fraction for three to 10 fractions and a total dose of 30–60 Gy were adopted in our institution. According to the experience of the Radiation Therapy Oncology Group 0236 guidelines, the dose constraints for the organs at risk (OAR) were implemented (2); When patients in the study received IMRT radiotherapy, the CT images and contours were directly transferred onto the 3D planning system (CMS XiO Treatment Planning System), and the total RT doses ranged from 30 to 60 Gy, while the daily doses ranged from 2.0 to 3.0 Gy. The OARs included the lungs, esophagus, heart, and spinal cord.

### Follow-up and evaluation

All patients were followed until death or December 2021. The patients were followed up every month for the first 3 months after their treatments and at 3-month intervals thereafter.

Local tumor progression-free survival (LTPFS) was defined as the interval from initial thermal ablation or helical tomotherapy to radiologic evidence of local tumor progression or the last follow-up date. OS was defined as the interval from initial ablation to death or the last follow-up date. Complications were recorded based on the Common Terminology Criteria for Adverse Events, v5.0. The major complications of the two groups [grade 3 or higher adverse event (AEs)] were compared.

### Statistical analysis

The baseline characteristics of the two groups were compared using chi-square test or *T*-test. Local tumor progression-free survival and overall survival rates were calculated using the Kaplan–Meier method with R (version 4.1.0). SPSS statistical software (version 24.0) was used for data analysis. The Cox proportional hazard model was used for univariate and multivariate analyses to determine the prognostic factors. Factors in the univariate analysis with *P <*0.1 were included in the multivariate analysis. The statistical significance of the multivariate analysis was defined as a *P*-value <0.05.

## Results

There were 106 out of the 110 patients included in the study at the end (63 patients in the ablation group and 43 patients in the helical tomotherapy group). Four patients were excluded from the final analysis (two due to ICC and two due to loss to follow-up). The mean follow-up time was 21.6 months (ranging from 1.3 to 87.8 months). The mean age was 55 years (ranging from 19 to 78 years). For treatments of primary liver cancer, 46 patients were treated with surgery, 52 patients had locoregional treatment, and 13 patients accepted systemic therapy in the thermal ablation group, and for the helical tomotherapy group, there were 41 patients who received surgery, 32 patients who went through locoregional treatment, and 15 patients who had systemic therapy. The baseline characteristics of the two groups were equivalent except for Child–Pugh grade (*P* = 0.048) and AFP (*P* = 0.008), as shown in **Table 1**.

**Table 1:** Baseline characteristics of patients with pulmonary metastases from hepatocellular carcinoma.

	Thermal ablation (*n* = 63)	Radiotherapy (*n* = 43)	*P*-value
Age (years)			0.617
<60	38	28	
≥60	25	15	
Gender			1.000
Female	5	3	
Male	58	40	
Tumor number			0.161
≤3	25	23	
>3	38	20	
Distribution of pulmonary metastatic tumors			0.926
Unilateral	24	16	
Bilateral	39	27	
Maximum tumor diameter, mm (mean ± SD)	17.5 ± 10.1	21.2 ± 16.8	0.205
Treatment of primary liver cancer			0.277
Surgery	46	41	
Locoregional treatment	52	32	
Systemic therapy	13	15	
History of previous pulmonary surgery			0.170
No	62	39	
Yes	1	4	
Lung metastasis			0.899
Metachronous	60	42	
Synchronous	3	1	
Extrapulmonary metastasis			0.052
No	58	34	
Yes	5	9	
Child–Pugh grade			**0.048**
A	52	41	
B	11	2	
Performance status			0.962
0	54	37	
1	9	6	
AFP (ng/ml)			**0.008**
<400	34	34	
≥400	29	9	

AFP, α-fetoprotein. The bold values are statistically significant with p <0.05.

### Local tumor progression-free survival

**Figure 2:**
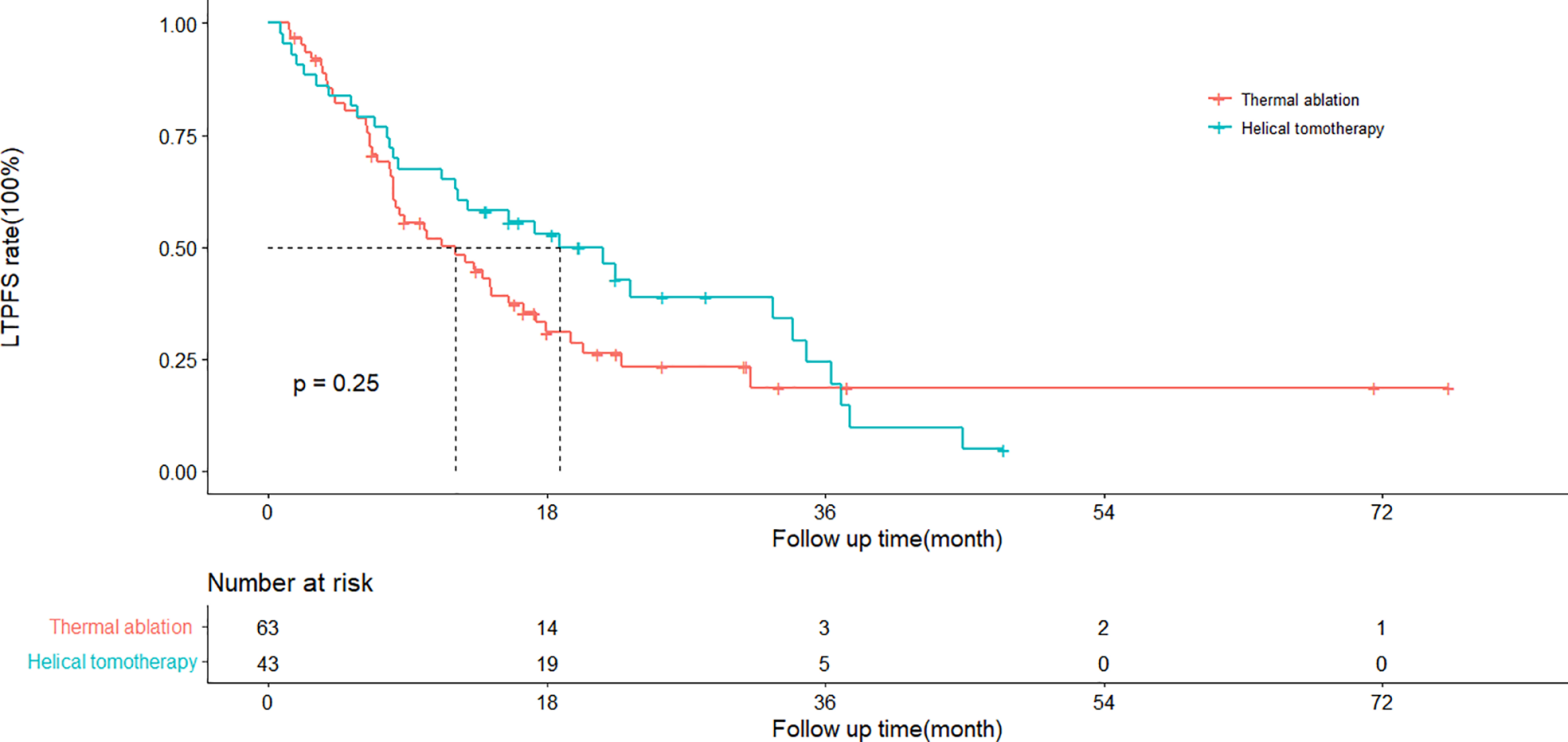
Kaplan–Meier curves of local tumor progression-free survival (LTPFS) in patients with pulmonary metastases from hepatocellular carcinoma who underwent thermal ablation or helical tomotherapy (thermal ablation group: *n* = 63, median LTPFS = 22.5 months; helical tomotherapy group: *n* = 43, median LTPFS = not reached; *P* = 0.13).

The cumulative 1- and 3-year LTPFS rates were 50 and 19% in the thermal ablation group and 65 and 25% in the helical tomotherapy group. The median LTPFS values of the thermal ablation and helical tomotherapy groups were 12.1 months (95%CI: 6.8–17.4 months) *vs*. 18.8 months (95%CI: 10.5–27.1 months) (*P* = 0.25), respectively (**Figure 2**). The multivariate analysis showed that serum AFP level (*P* = 0.003) was associated with LTPFS with statistical significance (**Table 2**).

**Table 2:** Univariate and multivariate analyses of prognostic factors for local tumor progression-free survival.

	Univariate model		Multivariate model	
	HR (95%CI)	*P*	HR (95%CI)	*P* ^a^
Gender				
Female				
Male	0.767 (0.343–1.716)	0.518		
Age (years)				
<60				
≥60	1.055 (0.652–1.707)	0.828		
Treatment of pulmonary metastatic tumors				
Thermal ablation				
Radiotherapy	0.761 (0.478–1.212)	0.251		
Lung metastasis				
Metachronous				
Synchronous	0.901 (0.220–3.685)	0.884		
History of previous pulmonary surgery				
No				
Yes	1.653 (0.601–4.548)	0.330		
Maximum tumor diameter (mm)				
<10				
≥10	2.113 (1.126–3.966)	0.020	1.733 (0.904–3.322)	0.097
Tumor number				
≤3				
>3	2.041 (1.277–3.262)	0.003	1.363 (0.801–2.318)	0.253
Distribution of pulmonary metastatic tumors				
Unilateral				
Bilateral	1.694 (1.046–2.745)	0.032	1.632 (0.974–2.814)	0.078
Extrapulmonary metastasis				
No				
Yes	1.251 (0.653–2.395)	0.500		
Child–Pugh grade				
A				
B	1.055 (0.505–2.203)	0.887		
AFP (ng/ml)				
<400				
≥400	2.353 (1.477–3.749)	<0.001	2.126 (1.298–3.482)	**0.003**

AFP, α-fetoprotein. ^a^Cox regression was used. The bold value is statistically significant with p < 0.05.

### Overall survival

**Figure 3:**
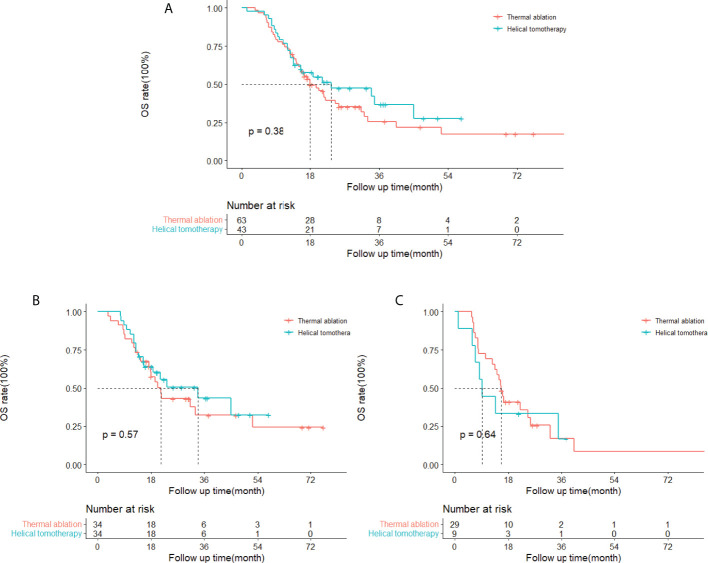
Kaplan–Meier curves of overall survival (OS) in patients with pulmonary metastases from hepatocellular carcinoma who underwent thermal ablation or helical tomotherapy. **(A)** Whole study population (thermal ablation group: *n* = 63, median OS = 18.0 months; helical tomotherapy group: *n* = 43, median OS = 23.4 months; *P* = 0.38). **(B)** Patients with low α-fetoprotein (AFP) level (AFP <400 ng/ml) (thermal ablation group: *n* = 34, median OS = 21.4 months; helical tomotherapy group: *n* = 34, median OS = 33.9 months; *P* = 0.57). **(C)** Patients with high AFP level (AFP≥400 ng/ml) (thermal ablation group: *n* = 29, median OS = 15.6 months; helical tomotherapy group: *n* = 9, median OS = 9.3 months; *P* = 0.64).

The cumulative 1- and 3-year OS rates were 75 and 26% in the thermal ablation group and 77 and 37% in the helical tomotherapy group. The median OS of the thermal ablation and helical tomotherapy groups were 18.0 months (95%CI: 12.6–23.3 months) and 23.4 months (95%CI: 4.4–42.5 months) (*P* = 0.38) (**Figure 3A**).

The results of the univariate and multivariate analysis indicated that tumor number (*P* = 0.016) and AFP (*P* = 0.010) were correlated with OS with statistical significance (**Table 3**). The subgroup analyses of OS in patients with different serum AFP levels showed that the median OS of patients with low AFP levels (<400 ng/ml) and high AFP levels (≥400 ng/ml) was 21.4 months (95%CI: 16.9–25.9 months) and 15.6 months (95%CI: 13.7–17.5 months), respectively, in the thermal ablation group but 33.9 months (95%CI: 12.4–55.4 months) and 9.3 months (95%CI: 6.6–12.1 months), respectively, in the helical tomotherapy group. The median OS of patients with low AFP levels or high AFP levels was not significantly different in the thermal ablation group compared with that in the helical tomotherapy group (*P* = 0.57 and *P* = 0.64, respectively) (**Figures 3B, C**).

**Table 3:** Univariate and multivariate analyses of prognostic factors for overall survival.

	Univariate model		Multivariate model	
	HR (95%CI)	*P*	HR (95%CI)	*P* ^a^
Gender				
Female				
Male	1.124 (0.449–2.816)	0.803		
Age (years)				
<60				
≥60	1.281 (0.781–2.103)	0.327		
Treatment of pulmonary metastatic tumors				
Thermal ablation				
Radiotherapy	0.800 (0.485–1.320)	0.382		
Lung metastasis				
Metachronous				
Synchronous	0.343 (0.047–2.484)	0.290		
History of previous pulmonary surgery				
No				
Yes	2.139 (0.768–5.955)	0.145		
Maximum tumor diameter (mm)				
<10				
≥10	1.321 (0.707–2.470)	0.383		
Tumor number				
≤3				
>3	2.602 (1.555–4.354)	<0.001	2.112 (1.153–3.868)	**0.016**
Distribution of pulmonary metastatic tumors				
Unilateral				
Bilateral	2.031 (1.190–3.467)	0.009	1.475 (0.779–2.795)	0.233
Extrapulmonary metastasis				
No				
Yes	1.775 (0.923–3.412)	0.085	1.828 (0.942–3.549)	0.074
Child–Pugh grade				
A				
B	1.400 (0.714–2.746)	0.327		
AFP (ng/ml)				
<400				
≥400	1.845 (1.135–3.000)	0.014	1.933 (1.169–3.196)	**0.010**

AFP, α-fetoprotein. ^a^Cox regression was used. The bold value is statistically significant with p < 0.05.

### Complications

There was no treatment-related death (grade 5 AE), grade 4 AE, and grade 3 AE in either the thermal ablation group or the helical tomotherapy group. Three patients in the thermal ablation group developed grade 2 AEs, including two patients (3.2%) with pneumothorax requiring thoracocentesis drainage and one patient (1.6%) with pleural effusion requiring thoracocentesis drainage. Grade 1 AEs, including mild pneumothorax (16 patients, 25.4%), mild pleural effusion (23 patients, 36.5%), and mild pneumonia (2 patients, 3.2%), were recorded in the thermal ablation group. Grade 2 radiation pneumonitis was recorded in two patients (4.7%) in the helical tomotherapy group. There was no grade 1 AE in the helical tomotherapy group (**Table 4**).

**Table 4:** Adverse events for two groups.

	Grade 2 AE	Grade 1 AE
Thermal ablation		
Pneumothorax	2	16
Pleural effusion	1	23
Pneumonitis	0	2
Total	3	41
Radiotherapy		
Radiation pneumonitis	2	0
Total	2	0

AE, adverse event.

## Discussion

The lung is one of the most frequent sites of extrapulmonary primary tumors’ metastatic spread, and systemic therapy has historically been regarded as the standard of care for this (6). However, some patients are not candidates for or unwilling to receive systemic therapy. For these patients, locoregional treatments, such as metastasectomy, radiotherapy, and thermal ablation, can be their choices. Although metastasectomy has traditionally been the mainstay of locoregional therapy, stereotactic ablative radiotherapy and thermal ablation are getting more and more accepted for they are non-invasive or less invasive, repeatable, safe, and others (6, 10). Percutaneous thermal ablation, such as RFA and MWA, has been demonstrated as a technically feasible and relatively safe treatment with impressive outcomes for patients with pulmonary metastases, and the majority of existing data is in the area of metastases colorectal carcinoma (6, 18, 19). In China, expert consensus recommended thermal ablation as a curative or palliative treatment for pulmonary metastases (13). Helical tomotherapy can deliver IMRT at a conformal high dose to a target while minimizing the high-dose radiation volume for the lung, the mean lung dose, and surrounding OARs, resulting in better dose uniformity, dose gradients, and protection for the organs at risk (20, 21). Due to the theoretical advantages of this technique, helical tomotherapy and its application in multiple tumor diseases, such as hepatocellular carcinoma and lung carcinoma, are becoming more prevalent (22–27). Our previous clinical studies had demonstrated its favorable tolerance, feasibility, and promising outcome for pulmonary metastasis from hepatocellular carcinoma (22, 27).

In this study, the 1- and 3-year LTPFS rates were 50 and 19% for the thermal ablation group and 65 and 25% for the helical tomotherapy group. The Kaplan–Meier method showed that the LTPFS rate of the thermal ablation group was a little lower than the helical tomotherapy group, but there was no statistical difference between the two groups (*P* = 0.25). A previous study (28) analyzing outcomes of percutaneous thermal ablation for pulmonary metastases from HCC showed that the 1- and 3-year LTPFS rates were 60.7 and 34.2%, which were better than the results of our study. This might be attributed to the patients in our study who owned a higher tumor burden, which meant more and larger pulmonary metastases. Another study showed that the 1- and 2-year progression-free survival (PFS) rates of RFA for pulmonary metastases from HCC were 59.7 and 28.2% (16). Jo et al. depicted that the 1-year PFS of helical tomotherapy for pulmonary oligometastases from hepatocellular carcinoma was 22.4%, and the median PFS was 4.9 months (17).

Hiraki et al. (29) retrospectively analyzed the results of percutaneous radiofrequency ablation for pulmonary metastases from hepatocellular carcinoma. They demonstrated that the 1- and 3-year overall survival rates were 87 and 57%, respectively, and the median survival time was 37.7 months. Another study using RFA for 26 patients with pulmonary metastases from HCC reported OS rates that were 88.5% at 1 year and 69.8% at 36 months (30). In this study, the 1- and 3-year OS rates were 75 and 26% for thermal ablation and 77 and 37% for helical tomotherapy. The OS rates of the previous research were higher than in our study. The possible explanations were that the present study included more patients, and the tumor diameters were larger than in the above-mentioned studies. A multicenter study examined the clinical outcomes of hypofractionated radiotherapy for pulmonary metastases from HCC and showed that the median OS was 16.3 months and the 1-year OS rate was 65.5% (17).

Natsuizaka et al. (31) investigated the prognostic factors for patients with extrahepatic metastases HCC and found that Child–Pugh class, metastasis to multiple extrahepatic organs, and serum AFP level were prognostic factors. Hiraki et al. (29) indicated that no viable intrahepatic recurrence, no liver cirrhosis, Child–Pugh A class, and serum AFP level lower than 10 ng/ml were associated with better survival for patients with pulmonary metastases from HCC undergoing percutaneous RFA. However, Kwon et al. (32) investigated patients with HCC accepting pulmonary metastasectomy and found that there were no independent prognostic factors. The present study found that survival after thermal ablation or helical tomotherapy of pulmonary metastases mainly relied on the tumor number and the serum AFP level. Patients with pulmonary metastases more than three (HR: 2.112, 95%CI: 1.153–3.868, *P* = 0.016) or a serum AFP level higher than 400 ng/ml (HR:1.933, 95%CI: 1.169–3.196, *P* = 0.010) correlated with poorer overall survival. Serum AFP level was also associated with LTPFS, with a higher AFP level (HR: 2.126, 95%CI: 1.298–3.482, *P* = 0.003) correlated with worse LTPFS. In addition, the subgroup analyses showed that thermal ablation and helical tomotherapy achieved similar OS in the low-AFP-level and the high-AFP-level groups, suggesting that these two treatments could acquire comparable outcomes.

The incidence of major complications was 0–25% (16, 28–30) in the thermal ablation group, most of which were pneumothorax requiring chest tube placement, and 0–12.1% (17, 33, 34) in the helical tomotherapy group. Ochiai et al. (34) compared the results of RFA and SBRT in solitary lung tumors and reported similar major complication rates for both groups (*P* > 0.999). The present study results were consistent with previous studies.

There are some limitations of this study. First, its retrospective nature was an important limitation. Some patients in this study lacked pathological confirmation and were diagnosed with clinical evidence, including radiological performance and serum AFP level, which the second limitation. Third, the sample size of this study was small, restricting the statistical power of the present study.

In conclusion, thermal ablation and helical tomotherapy provided similar local tumor progression-free survival and overall survival for pulmonary metastases from hepatocellular carcinoma with equal safety.

## Data availability statement

The original contributions presented in the study are included in the article/supplementary material. Further inquiries can be directed to the corresponding authors.

## Ethics statement

The studies involving human participants were reviewed and approved by the Institutional Review Boards of the Zhongshan Hospital, Fudan University. Written informed consent for participation was not required for this study in accordance with the national legislation and the institutional requirements.

## Author contributions

LL and JH conceived and designed the project. ZY and ZZ provided administrative support. FW, SF, QS, and DZ collected the data. FW, SF, QS, and HyS analyzed and interpreted the data. All authors contributed to the article and approved the submitted version.

## Funding

This study has received funding from the Shanghai Municipal Health Commission (no. 201940409).

## Conflict of interest

The authors declare that the research was conducted in the absence of any commercial or financial relationships that could be construed as a potential conflict of interest.

## Publisher’s note

All claims expressed in this article are solely those of the authors and do not necessarily represent those of their affiliated organizations, or those of the publisher, the editors and the reviewers. Any product that may be evaluated in this article, or claim that may be made by its manufacturer, is not guaranteed or endorsed by the publisher.
