# Corrigendum: Comparison of clinical outcomes between cone beam CT-guided thermal ablation and helical tomotherapy in pulmonary metastases from hepatocellular carcinoma

**DOI:** 10.3389/fonc.2022.1038584

**Published:** 2022-10-06

**Authors:** Feihang Wang, Shaonan Fan, Qin Shi, Danyang Zhao, Huiyi Sun, Yav Sothea, Mengfei Wu, Huadan Song, Yi Chen, Jiemin Cheng, Zhaochong Zeng, Zhiping Yan, Jian He, Lingxiao Liu

**Affiliations:** ^1^ Department of Interventional Radiology, Zhongshan Hospital, Fudan University, Shanghai, China; ^2^ Shanghai Institute of Medical Imaging, Fudan University, Shanghai, China; ^3^ National Clinical Research Center for Interventional Medicine, Zhongshan Hospital, Fudan University, Shanghai, China; ^4^ Department of Radiation Oncology, Zhongshan Hospital, Fudan University, Shanghai, China; ^5^ Shanghai Medical College, Fudan University, Shanghai, China; ^6^ Department of Computed Tomography (CT) and Magnetic Resonance Imaging (MRI), Third Hospital of Hebei Medical University, Shijiazhuang, China; ^7^ Department of Radiology, Xinhua Hospital, Shanghai Jiao Tong University School of Medicine, Shanghai, China

**Keywords:** pulmonary metastases, hepatocellular carcinoma, thermal ablation, helical tomotherapy, comparison

In the published article, there was an error in affiliation of Zhaochong Zeng and Jian He. Instead of” **Zhaochong Zeng^2^**”and “Jian He^2^*”, the Shanghai Institute of Medical Imaging, Fudan University, Shanghai, China, it should be “**Zhaochong Zeng^4^**” and “Jian He^4^*”, the Department of Radiation Oncology, Zhongshan Hospital, Fudan University, Shanghai, China.

The authors apologize for this error and state that this does not change the scientific conclusions of the article in any way. The original article has been updated.

## Publisher’s note

All claims expressed in this article are solely those of the authors and do not necessarily represent those of their affiliated organizations, or those of the publisher, the editors and the reviewers. Any product that may be evaluated in this article, or claim that may be made by its manufacturer, is not guaranteed or endorsed by the publisher.

